# Evaluation of the efficacy of the Lentulo spiral filler operated at four different speeds and with two different techniques in cadaveric canine teeth of dogs

**DOI:** 10.3389/fvets.2023.1295306

**Published:** 2023-11-02

**Authors:** Haley Carlson, Jennifer Montebello, Bonnie Lee, Aaron Rendahl, Stephanie Goldschmidt

**Affiliations:** ^1^BluePearl Pet Hospital, Arden Hills, MN, United States; ^2^Department of Veterinary Clinical Sciences, University of Minnesota, St. Paul, MN, United States

**Keywords:** Lentulo spiral filler, obturation, root canal therapy, canine tooth, veterinary endodontics, small animal dentistry, AH Plus Jet, sealer cement

## Abstract

This study evaluated the effect of filling technique and rotational speed on the efficacy of sealer cement placement using the Lentulo spiral filler. Cadaveric maxillary and mandibular canine teeth (n = 74) from age and breed matched fresh-frozen adult canine cadaver heads were utilized. Following routine mechano-chemical preparation, teeth were randomly divided into 8 treatment groups with varying rotational speeds [250–2,000 revolutions per minute (RPM)] and fill techniques (backfill technique versus pumping technique). The quality of the sealer cement fill was evaluated radiographically before and after master gutta percha cone placement. Percentage of voids present in the apical, midbody, and coronal portions of the tooth were subjectively scored as: no voids present, voids present in less than 25% of the area, voids present in 25–50% of the area, voids present in greater than 50% of the area. The apex was also scored as underfilled, adequately filled, or overfilled. Operating the Lentulo spiral filler at 2,000 RPM resulted in the best quality fill regardless of filling technique. The pumping technique with placement of the master gutta percha cone had a higher probability of success as compared to the backfill technique, but this finding lacked significance. Regardless of speed or technique, the apical region had the highest quality of fill. The addition of the master gutta percha cone improved the quality of fill. Therefore, we recommend using the Lentulo spiral filler at higher speeds with a pumping technique (followed by addition of a master gutta percha cone) to improve the quality of epoxy resin-based sealer cement placement.

## Introduction

Successful root canal therapy (RCT) depends on both the ability to properly disinfect the pulp cavity and the quality of the seal established during obturation and restoration (1–3). Following mechano-chemical preparation, proper obturation prevents the re-introduction and growth of bacteria in the endodontic system, which allows for periapical healing (4, 5). Obturation is primarily performed by placement of a master gutta percha (GP) cone (6). However, sealer cement is also required to achieve appropriate obturation because master GP cones cannot adhere to dentinal walls, and rarely, completely fill the prepared canal, particularly in veterinary dentistry (6, 7). Sealer cement is essential for proper obturation of the canal as it fills voids not filled by the GP cone, and ideally, also creates a primary monoblock with the cone to prevent re-colonization of bacteria (7, 8).

Voids in the endodontic canal, especially in the apical third of the tooth, increase the chance for bacterial contamination and resultant RCT failure (1, 3). Thus, proper placement of the sealer cement, especially in this region, is critical. In fact, most endodontic studies rely on dental radiographs to evaluate optimal fill of the apex (5, 9–11). Alternatively, apical microleakage and clinical studies have also been utilized to evaluate the quality of the apical seal and subsequent risk of endodontic failure (12–14).

**Figure 1:**
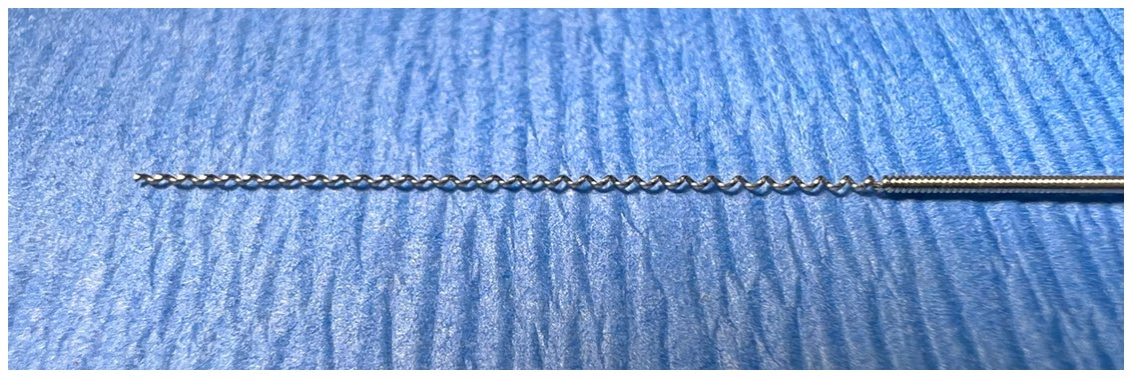
Spiral working end for sealer cement delivery on the Lentulo spiral filler.

There are a variety of instruments and techniques available to administer sealer cements (2, 15). A commonly used instrument, especially in veterinary dentistry, is the Lentulo spiral filler (Figure 1) (2). Yet, there is limited published literature on the proper speed or technique to maximize success with this instrument. Two veterinary studies performing endodontic procedures describe operating the Lentulo spiral filler on a low-speed handpiece with a 10:1 reduction gear (16, 17). No specific speed or technique is discussed (16, 17). To the authors’ knowledge, there is only a single veterinary reference that describes the technique for proper use, which describes using lower speeds (no revolutions per minute (RPM) listed) and withdrawing the Lentulo spiral filler from the root canal in a clockwise, pumping motion (18).

There is also no standard for Lentulo spiral filler use in human dentistry (15). In research publications, most do not list the speed or technique (4, 19–21) that was utilized. When speed is listed, a range of speeds from 300 RPM to 2,000 RPM are reported (1, 11, 22–25).

Furthermore, multiple fill techniques are described in the human literature with no data on resultant differences in clinical outcome. Two main techniques were commonly identified on literature review. The first described placing the rotating Lentulo spiral filler apically until backfill of flowable sealer cement is seen at the access site. Once backfill is seen, the instrument is slowly withdrawn from the canal with continued rotation (1, 5, 9, 22, 26). The second technique described placing the rotating Lentulo spiral filler at the apical region and applying slight pumping motion prior to withdrawing completely (23, 25). Moving forward, we refer to these two techniques as the backfill and pumping method, respectively.

The lack of clarity surrounding the proper use of the Lentulo spiral filler makes clinical use of this instrument arduous. The aim of this study is to compare the efficacy of sealer cement placement using the Lentulo spiral filler at different speeds and filling techniques. Our hypothesis is that a pumping method at higher speeds will provide the most complete fill with minimal voids in the canal, especially at the apical third.

## Materials and methods

### Mechano-chemical root canal preparation

Mandibular and maxillary canine teeth from fresh-frozen adult canine heads that had been euthanized for reasons unrelated to this study were utilized. All canine heads were sourced from a single research colony and were the same breed and age (approximately 1 year old) and had closed apices. This study did not involve the use of live animals; thus, we were exempt from IACUC ethical approval. The teeth remained *in situ* for the study to best replicate a clinical situation. Teeth were crown amputated with a cross-cut fissure (701 L) bur, leaving 5 millimeters (mm) of clinical crown remaining above the gingiva, allowing for straight-line access to the apices.

Prior to obturation, the teeth were assessed grossly, radiographically, and with cone beam computed tomography (CBCT) to ensure lack of structural defects and homogeneity in pulp cavity width among sample groups. Pulp cavities were measured on the CBCT in the mesial-distal oblique plane and the coronal plane by a single evaluator (SG). The volume of each pulp cavity was calculated using the formula for the volume of a cone, $V=1/3\left(\pi r^{2}h\right)$ , as previously described (27). In this equation *V* = volume, *r* = radius of the pulp cavity, and *h* = height of the pulp cavity. The radius was measured on the mesial-distal oblique view, as this is most similar to a dental radiograph.

Following volumetric analysis, routine RCT was performed on each tooth with a 50 mm rotary file (LightSpeed LSX Files; Kerr Corporation, Orange, CA) on a rotary motor (ProMark Endodontic Motor; Dentsply Tulsa Dental Specialties, Johnson City, TN) running at 1500 RPM. Canals were lavaged with sodium chloride between each successive file size. Most were filed to a master apical rotary (MAR) size of 140. Ethylenediaminetetraacetic acid (EDTA) was placed in the canal for 1 min after instrumentation was complete. A final sterile saline rinse was performed, and the canals were dried with paper points (Absorbent Paper Points; Shipps Dental and Specialty Products, Marana, AZ). The master GP cone size for each canal was determined by using MAR minus 50 “rule,” which states to subtract 50 from the MAR to obtain the appropriately sized standard master GP cone to be placed retrograde (12, 13). A trial fit with the calculated master GP cone (Gutta Percha Points; Shipps Dental and Specialty Products, Marana, AZ) was performed prior to obturation to ensure that the cone snuggly fit into the apical portion of the canal and was at working length. Adjustments to master GP cone size were made as needed based on the trial fit radiograph using clinical judgment. All mechano-chemical preparation for the RCTs were performed by two residents in veterinary dentistry and oral surgery (HC, BL).

### Obturation

Prior to obturation, the teeth were randomly distributed into 8 study groups using a random number generator. An epoxy resin-based sealer cement (AH Plus Jet; Dentsply DeTrey, Konstanz, Germany) was utilized in all study groups with a size 40, 60 mm long Lentulo spiral filler (Spiral Paste Filler; Henry Schein Inc., Melville, NY). A rotary motor was used to control speed. Four different speeds and two fill techniques were evaluated (Table 1).

**Table 1:** Outline of study groups divided into speeds and techniques performed.

Group	Speed	Technique
1A	250 RPM	Pumping
1B	250 RPM	Backfill
2A	500 RPM	Pumping
2B	500 RPM	Backfill
3A	1,000 RPM	Pumping
3B	1,000 RPM	Backfill
4A	2000 RPM	Pumping
4B	2000 RPM	Backfill

For the backfill technique, the Lentulo spiral filler was prefilled with sealer cement and placed at the most apical aspect of the pulp cavity (working length). It was rotated in a clockwise rotation at the dedicated speed at the apex for 120 s as sealer cement was continuously applied to the Lentulo spiral filler at the access site. The access was evaluated for visual presence of bubbles. If bubbles were seen following 120 s, then the Lentulo spiral filler was spun at the apex for an additional 30 s past when no bubbles were grossly visualized. The clinician then slowly removed the Lentulo spiral filler from the canal while it continued to rotate. The total time spent at the apex and the time for removal of the Lentulo from the canal was recorded.

For the pumping technique, the Lentulo spiral filler was used at the dedicated speed to slowly pump the continuously applied AH Plus sealer cement to the most apical aspect of the pulp cavity (working length) and out of the canal 5 times, regardless of the appearance of the sealer cement at the access site. The total time to complete obturation was recorded.

Obturation was performed by a single board-certified veterinary dentist (SG) to ensure consistency. A lateral radiograph was performed of each tooth immediately following AH Plus sealer cement application using the bisecting angle technique. Following radiographs, the previously determined master GP cones were placed and the teeth were restored with glass ionomer (Ionoseal; Voco, Cuxhaven, Germany). All cavity preparations and restorations were performed by a resident in dentistry and oral surgery (BL). Radiographs were performed immediately following restorative placement.

### Radiographic evaluation

**Figure 2:**
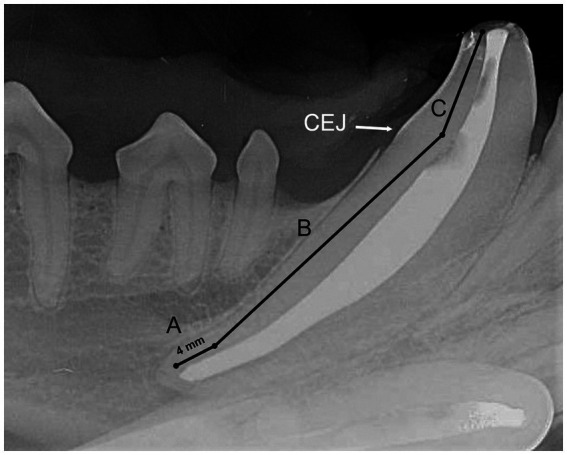
Radiographic image of a canine tooth following sealer cement placement that demonstrates the locations of evaluation for the apical **(A)**, midbody **(B)**, and coronal **(C)** sections.

**Figure 3:**
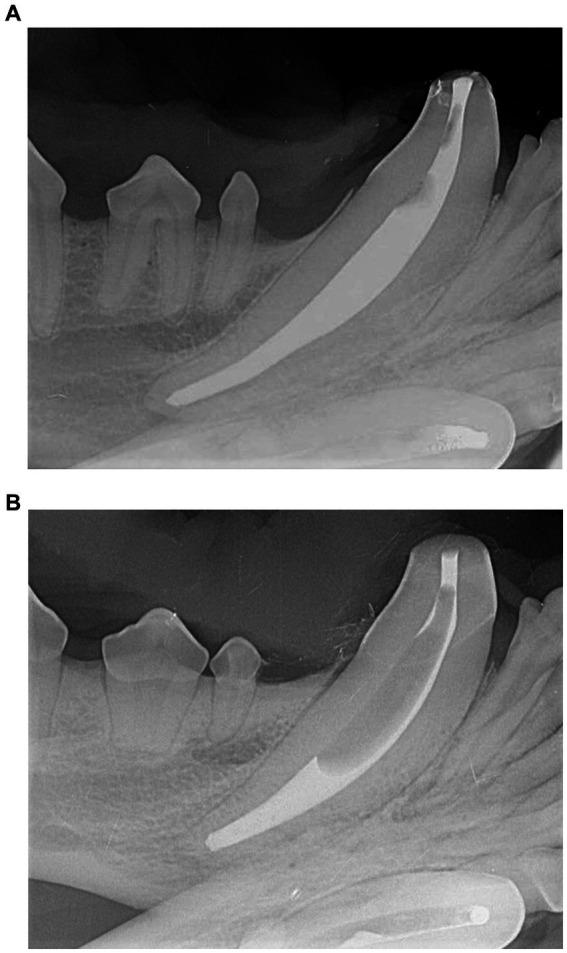
Example of clinical success scoring after sealer cement +/− GP cone placement. Example of scores 1–3 in a single tooth **(A)**. Specifically, no voids (score 1) in the apical region, <25% voids (score 2) in the midbody region, and 25–50% voids (score 3) in the coronal region. Example of >50% voids (score 4) **(B)** in the midbody and coronal region.

Radiographic evaluation was performed by a board-certified veterinary dentist and a resident in dentistry and oral surgery (SG, HC). The quality of fill was graded separately for sealer cement only and sealer cement + master GP cone. The quality of the fill was graded for each tooth location separately, namely apical, midbody, and coronal (Figure 2). The apical section was defined as 4 mm from the most apical portion of pulp. The midbody was the section of the pulp cavity from the apical portion to the cementoenamel junction (CEJ). The coronal section included the region of the pulp cavity that was coronal to the CEJ. Each location was given a numerical score using a previously described grading scheme (23) (Table 2, Figure 3). The apical section also received an additional numerical score based on if the fill was adequate, underfilled, or overfilled using modified Coll and Sadrian criteria (28, 29) (Table 3). Lastly, teeth were overall scored as having a homogenous or inhomogeneous fill. All scores were reached by consensus.

**Table 2:** Scoring system on quality of fill for coronal, midbody, and apical section of each tooth.

Score	Description
1	No voids present
2	Voids present in less than 25% of the root canal area
3	Voids present in between 25–50% of the root canal area
4	Voids present in greater than 50% of the root canal area

**Table 3:** Scoring system for evaluation of apical fill for each tooth.

Score	Description	Definition
1	Underfilled	Filled more than 2 mm short of the apex
2	Adequately filled	Filled up to 2 mm short of the apex
3	Overfilled	Filled past the apex

### Statistical analysis

To explore relationships between technique, RPM, location (apical, midbody, coronal), placement of the master GP cone, tooth type (maxillary versus mandibular), and the probability of having a successful fill (quality of 1), a logistic generalized estimating equation model was fit with the mentioned variables as predictors, including all interactions with cone placement and tooth as a repeated measure with exchangeable structure. Odds ratios with 95% confidence intervals and *p*-values are reported. The average probabilities for combinations of interest are also computed as average probabilities across all other variables.

To explore the probability of having adequate fill, a similar model was fit with technique, RPM, placement of the GP cone, and tooth type as predictors; location was not included as fill is assessed overall, not by location. To explore the probability of being homogeneous, however, the number of events was too small to model using this method. For within-tooth variables (GP cone placement), McNemar’s test was used. For between-tooth variables, the count of total homogenous results for each tooth was calculated and assessed using Wilcoxon’s test for technique and tooth type and using Kendall’s test for RPM.

Several covariates were also explored, including CBCT measurements of pulp cavity widths of the coronal region in long and short axis (which were used to calculate the area of ellipse) and the pulp cavity widths and heights (to the CEJ and to the most coronal aspect) in mesial-distal oblique plane. First, to check the randomization of teeth to treatment (technique and RPM combination), ANOVA models were fit with each potential covariate as the response and treatment as the predictor. Similarly, to explore the relationship of these covariates with tooth type, ANOVA models were also fit with tooth type as the predictor variable. These covariates, along with the time to perform the treatment, were then added to the logistic model for quality and any significant relationships were explored.

Finally, to explore the relationship between technique, RPM, tooth type, and the time to perform the treatment, an ANOVA model was fit with time as the response variable.

For all results, *p* < 0.05 was considered statistically significant.

## Results

Treatment and initial radiographic analysis were performed in 74 cadaveric maxillary and mandibular canine teeth. Two teeth were excluded from analysis due to rotary file separation within the canal during mechanical debridement (*n* = 1) and inability to reach the apex during operation of the Lentulo spiral filler (*n* = 1). Results from all interactions are shown in Supplementary Table 1.

### Effect of speed and technique on quality of the sealer cement fill

**Figure 4:**
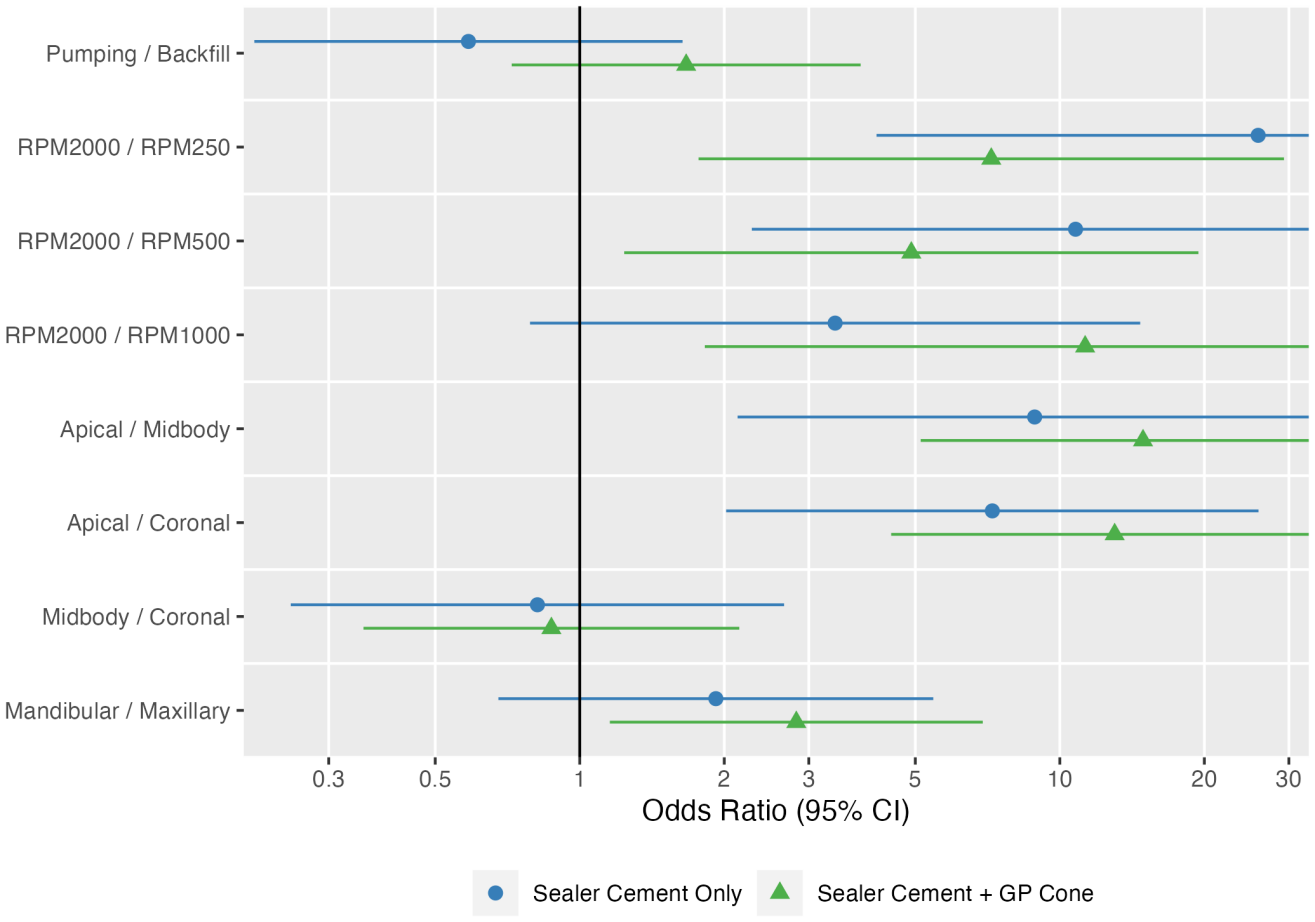
Estimated odds ratios with 95% confidence intervals for technique, speed, individual tooth region (apical, middle, coronal), and tooth type, shown separately as sealer cement only and sealer cement + master cone. The point and line represent the odds ratio and 95% confidence interval from the logistic model.

**Figure 5:**
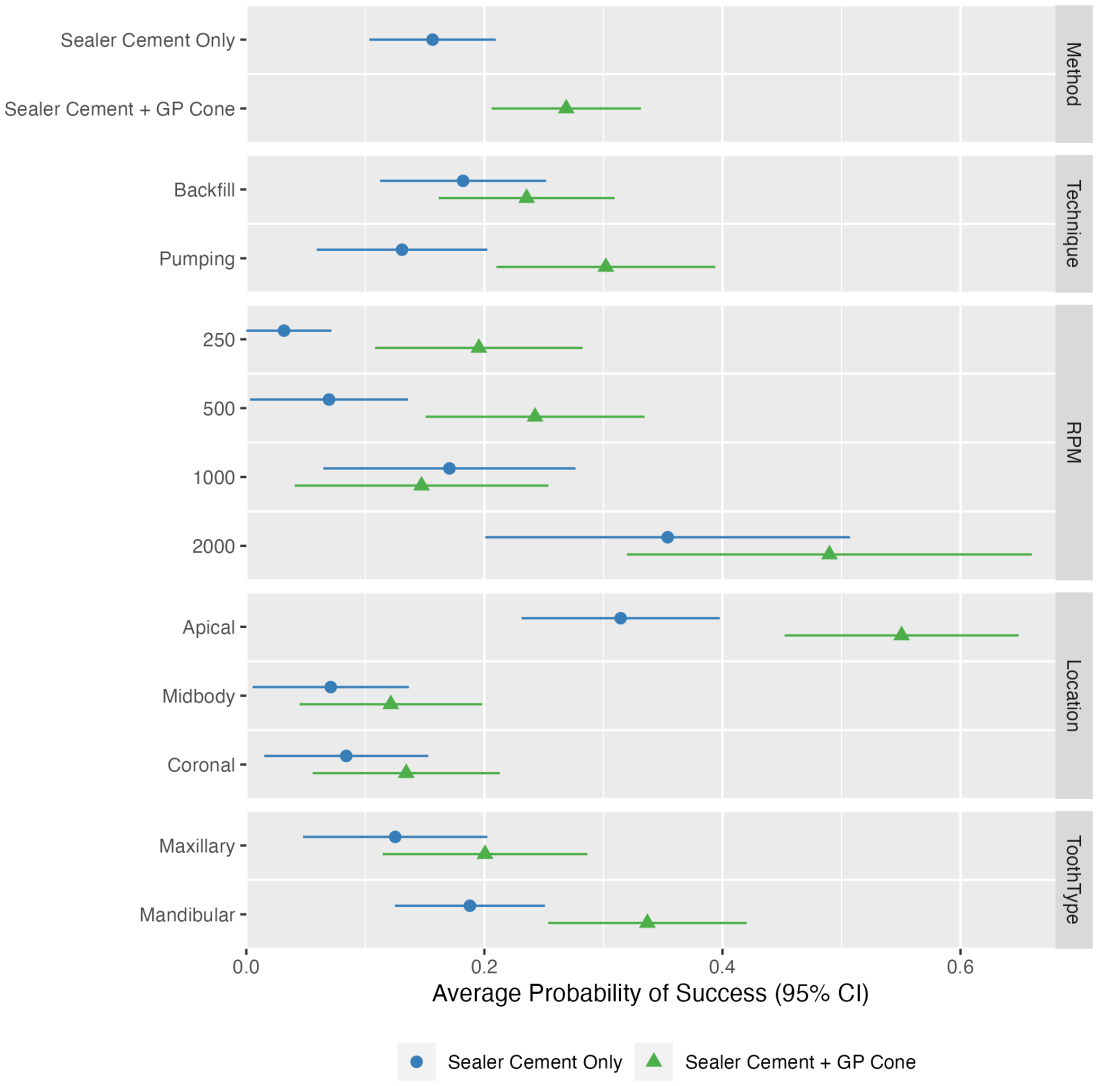
Estimated probabilities of success for all possible combinations of technique, speed, individual tooth region (apical, middle, coronal), tooth type, and cone placement (sealer cement only versus sealer cement + master cone). The bar represents the observed proportion of success; the point and line represent the estimated probability and 95% confidence interval from the logistic model. Note the higher probability of success in quality of fill at speeds of 2,000 RPM compared to other speeds and higher probability of success in the apical region compared to other regions. Additionally, a higher probability of success is noted following placement of the master cone.

Higher speeds were significantly (*p* < 0.0001) associated with better fill (Figures 4, 5). An RPM of 2,000 had the highest probability of achieving quality of fill (no voids; score of 1), with an overall estimated odds ratio of 6.2 (95% CI: 1.4, 27) compared with 1,000 RPM, 7.3 (95% CI: 2.0, 25.9) compared with 500 RPM, and 13.7 (3.4, 54.1) when compared with 250 RPM. The average estimated success rate at 2,000 RPM was 42.2%, compared with 15.9% at 1,000 RPM, 15.6% at 500 RPM, and 11.4% at 250 RPM.

Overall, the technique utilized (pumping vs. backfill) was not significantly (*p* = 0.98) associated with quality of the fill. The pumping technique had an overall estimated odds ratio of 0.99 (95% CI: 0.44, 2.21) compared with the backfill technique. However, there was a significant interaction between technique and cone placement (*p* = 0.034); when the cone was placed, pumping was associated with more success with an odds ratio of 1.67 (95% CI: 0.72, 3.85), in contrast with before the cone when the odds ratio was 0.59 (95% CI: 0.21, 1.64).

**Figure 6:**
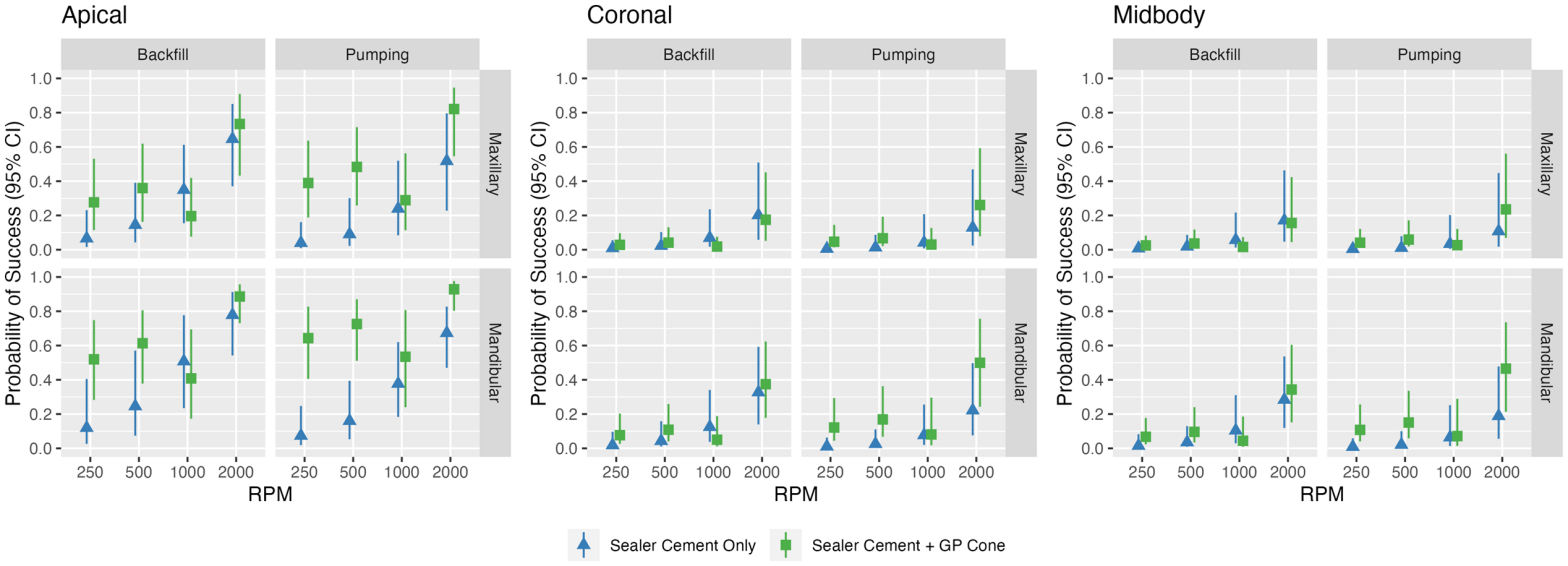
Odds ratio and estimated probabilities of success for all possible combinations of technique and speed for different tooth regions. The bar represents the observed proportion of success; the point and line represent the estimated probability and 95% confidence interval from the logistic model.

Location (apical, midbody, coronal) was also significantly (*p* < 0.0001) associated with quality of fill. The probability of success was highest in the apical location with an estimated odds ratio of 11.5 (95% CI: 3.9, 33.7) compared with midbody location and 9.7 (95% CI: 3.7, 25.5) compared with coronal location; the average estimated success rates were 43.3, 9.6, and 10.9%, respectively (Figure 6).

The addition of a master GP cone (*p* = 0.001) following sealer cement increased the quality of the fill. The probability of success was higher with an odds ratio of 2.6 (95% CI: 1.5, 4.7) compared with sealer cement; the average estimated success rates were 26.9 and 15.6%, respectively. The probability of success was also estimated to be higher in mandibular teeth than maxillary teeth with an odds ratio of 2.33 (95% CI: 0.99, 5.49), though this did not reach statistical significance (*p* = 0.053); the average estimated success rates were 26.2 and 16.3%, respectively.

### Evaluation of covariates

**Figure 7:**
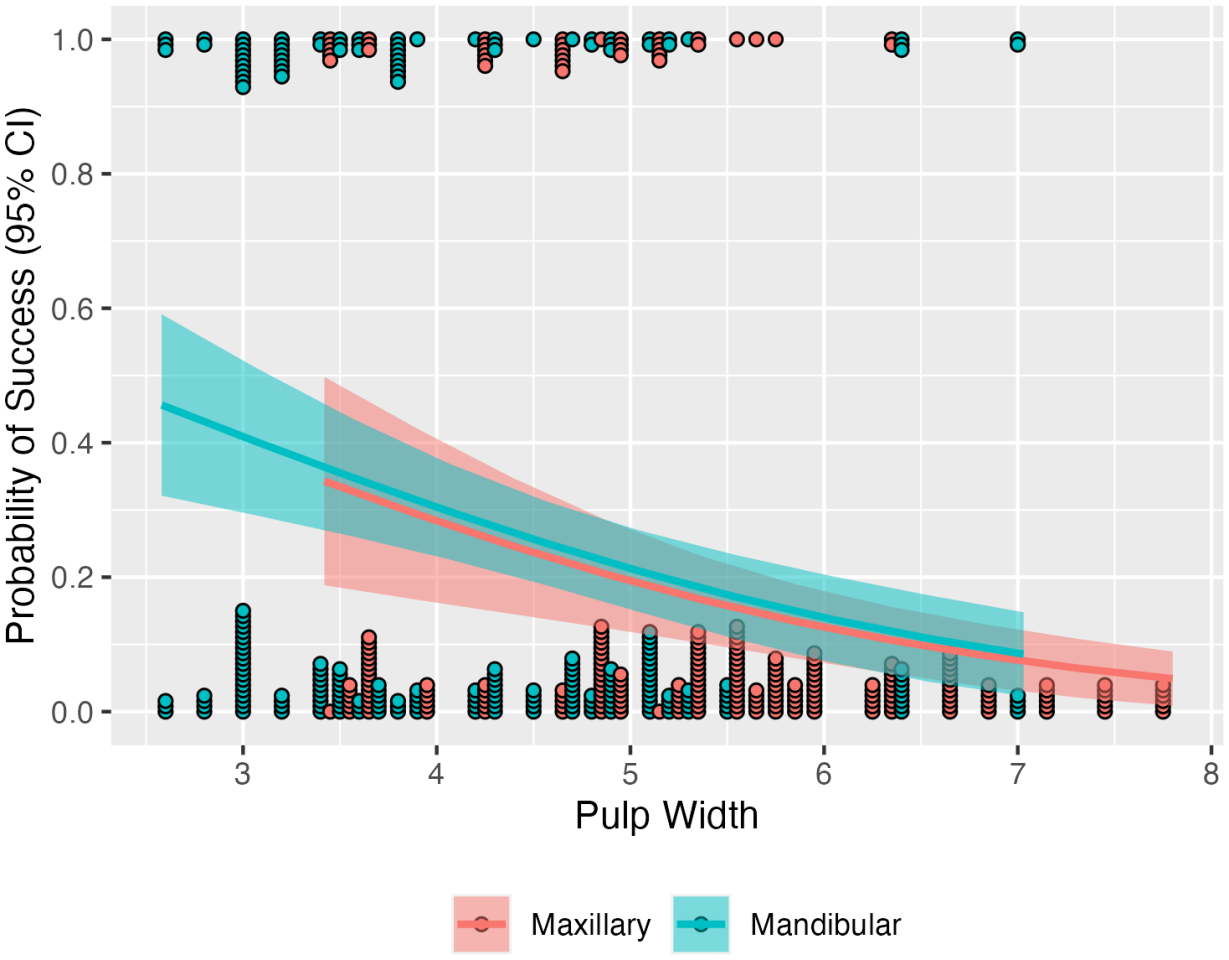
Estimated probability of success by pulp width and tooth type, with 95% confidence intervals. Points indicate individual teeth of each type that either were successful (those on the top) or not (those on the bottom). The probability of success decreases as pulp width increases, similarly for both types of teeth.

No tooth specific covariates (measurements of the pulp cavity widths and heights in multiple CBCT planes) were significantly associated with treatment type (all *p* > 0.15) and only pulp width was significantly different between maxillary and mandibular (*p* < 0.0001) teeth with a mean of 5.37 mm (95% CI: 5.00, 5.73) for maxillary teeth and a mean of 4.21 mm (95% CI: 3.84, 4.58) for mandibular teeth. Additionally, when all covariates, as well as the time to perform the treatment, were added to the logistic model for success, only pulp width was significant (*p* < 0.05) with a standardized odds ratio of 0.27 (95% CI: 0.11, 0.64). To explore further how success depends on pulp width and tooth type, the logistic model was refit with only pulp width added and the estimated average probability was computed across the range pulp widths (Figure 7). This showed that the success rate was associated with pulp width, not tooth type; the estimated average probability was very similar between the tooth types, while it did change with pulp width from an average success rate of 39.9% at 3 mm to 8.15% at 7 mm.

### Effect of speed and technique on fill at the apex

With sealer cement only, 28% (21/74) were underfilled, 72% (53/74) were adequate, and none were overfilled. Once the GP cone was placed 8.2% (6/73) were underfilled, 86% (63/73) were adequate, and 5.5% (4/73) were overfilled. Placement of the GP cone was significantly (*p* = 0.015) associated with the apical fill. The proportion of both adequate fill and overfilled increased with placement of the GP cone. No other factors influenced the likelihood of having an underfilled, adequate, or overfilled apex.

### Effect of speed and technique on homogeneity of root canal fill

The only statistically significant factor on homogeneity was speed (*p* = 0.0013). Homogeneity increased with speed from 0% (0/40) and 2.5% (1/40) at 250 and 500 RPM to 5.9% (2/34) and 21% (7/34) at 1,000 and 2,000 RPM.

### Effect of speed and technique on time

The time to perform the treatment was longer for the backfill technique than the pumping technique (*p* = 0.011) with estimated average times of 211 (95% CI: 199, 222) and 189 s (95% CI: 177, 201), respectively. The time also increased with speed (*p* = 0.0029) from 182 (95% CI: 166, 197) and 188 s (95% CI: 172, 204) at 250 and 500 RPM to 206 (95% CI: 188, 223) and 224 s (95% CI: 206, 241) at 1,000 and 2,000 RPM. The association with tooth type was not significant (*p* = 0.94).

## Discussion

This study revealed that speed was the most impactful factor in the quality of fill when utilizing the Lentulo spiral filler. Higher rotary speeds of 2,000 RPM for administering an epoxy resin-based sealer cement resulted in fewer voids compared to speeds of 250 RPM, 500 RPM, and 1,000 RPM.

To the authors’ knowledge, the current study is the first in veterinary literature to evaluate the appropriate speed to use the Lentulo spiral filler during RCT obturation. The present study utilized an endodontic motor that can be set to specific speeds. Clinically, some may not have access to this type of endodontic motor, rather the Lentulo spiral filler is operated on a reduction contra-angle attached to a low-speed handpiece on an air-driven dental unit. Most low-speed motors run at speeds ranging from 5,000 to 40,000 RPM with speeds of 5,000 to 20,000 RPM being most common (30–34). Reduction contra-angles are labeled as a reduction gear ratio, indicating the factor by which the speed of the low-speed motor is decreased by the contra-angle attachment. For example, a 4:1 reduction contra-angle makes a low-speed motor that runs at 20,000 RPM, 4 times slower (equaling 5,000 RPM). Given a low-speed motor that operates at 20,000 RPM, a 10:1 reduction angle would reduce the speed to 2,000 RPM. With the results from the current study, this would be the recommended contra-angle to utilize. However, the clinician may need to adjust based on the speed of their low-speed motor. Additionally, speeds greater than 2,000 RPM were not evaluated in this study, so it is unknown if speeds greater than 2,000 RPM would result in even fewer voids.

Rare human studies have reported on the speed of the Lentulo spiral filler as a factor impacting successful obturation. Compared to different methods of obturation, Atmeh et al. demonstrated a similar fill achieved with a Lentulo spiral filler operated at 2,000 RPM (25). Other studies showed that different methods of obturation were superior to a Lentulo spiral filler operated at 500 RPM and 1,000 RPM, depending on the study (1, 10, 24). These results are consistent with the findings of the current study when a Lentulo spiral filler operated at 2,000 RPM had the greatest odds ratio of a quality of fill.

Deonizio et al. is one of the only studies in human literature that directly evaluated the quality of fill with operation of the Lentulo spiral filler at different speeds (5). Higher speeds of 15,000 RPM were noted to provide the best quality of fill in the apical regions while speeds of 5,000 RPM were more effective for filling the coronal and middle portions of the pulp cavity (5). These results are also consistent with the findings of the current study demonstrating that the operational speed of the Lentulo spiral filler was associated with the quality of fill. However, direct comparison is limited as the speeds used were almost 10 times those of the current study.

One of the concerns of using the Lentulo spiral filler at higher speeds is the theoretical risk of apical extrusion based on human literature. Human studies have shown that higher speeds (10,000, and 20,000 RPM) have an increased risk compared to 5,000 RPM (35). Extrusion of sealer cement during obturation may have negative impacts on RCT success. Some have argued that these effects may even be more significant than underfilling of the canal (18, 36–38). The possibility of a decrease in clinical success due to extrusion is likely related to the inflammation of the periapical tissues that occurs in response to certain sealer cement materials (39, 40). Yet, direct comparison to human literature is cautioned due to anatomical differences of the apex. To the authors’ knowledge there is no veterinary literature linking the speed of Lentulo spiral filler use to the risk of extrusion, nor the effect of extrusion on RCT success/periapical tissues. Of interest, within the current canine cadaveric study, higher speeds did not result in an increased risk of extrusion of sealer cement. Yet, this risk is also significantly minimized in both a cadaveric and clinical setting with a mature closed apex. In an immature tooth the speed of the lentulo may directly affect the risk of overfill, and should be used with caution.

In the present study, we chose to evaluate the fill of the entire pulp cavity and then also in the coronal, middle, and apical regions separately. We defined the apical region as 4 mm within the most apical portion of the pulp cavity. This ensured that the portion of the pulp cavity containing the apical delta was included within the apical region among a variety of specimens. The apical delta contains multiple apical ramifications connecting the pulp to surrounding tissue (6, 41). While apical delta height has been previously measured as 1–3 mm from the external apex of the tooth, some teeth can have an apical delta height > 3 mm (2, 18, 41) and dentinal thickness varies among specimens especially in young patients. Thus to ensure inclusion of the delta we measured from the visible portion of the pulp rather than apex. Furthermore, 74% of accessory canals are found in the apical third of the endodontic canal (2, 36). We elected to include only 4 mm of radiographically visible pulp tissue rather than a third of the pulp cavity in an effort to focus on the region most likely to be clinically impactful for RCT failure across a variety of specimens. Based on the current body of literature this is unlikely to be >4 mm of visible pulp, if even that. However, thorough data on the true extent of lateral canals and anatomical variation in the apical delta in dogs is lacking, and this may have biased the data interpretation.

It was found that the apex had the highest probability of having quality of fill in comparison to the midbody and coronal regions, regardless of speed or technique. This is likely due to the nature of fill techniques being centered around delivery of sealer cement to the apex. To ensure quality fill of the entirety of the canal, more time likely needs to be spent at the coronal portion. This is likely to be of even more importance in larger pulp cavity sizes as seen in the current study. This study also evaluated the effect of technique on fill. While there was no overall statistically significant difference between the two methods, there was evidence that the probability of success with the pumping technique compared to the backfill technique increased when the cone was applied.

As expected, the quality of fill was further improved by the addition of the master GP cone. Overall, the number of adequately filled canals increased by 14% following placement of the master GP cone. Furthermore, 15/17 (88.24%) teeth treated with the Lentulo spiral filler operated at 2,000 RPM (regardless of the filling technique) and placement of the master GP cone had no evidence of voids in the apical section. The remaining two teeth in this group showed voids of less than 25% in the apical section after addition of the master GP cone. These findings support the importance of using a master GP cone in addition to sealer cement to achieve an adequate fill.

A homogenous fill throughout the entirety of the endodontic canal is essential for reducing the number of voids throughout the canal, and subsequently, the risk of re-colonization of bacteria. While radiographic assessment does not represent the full picture of clinical success, a lack in uniformity could result in failure later (2, 16). In this study, homogeneity was noted to increase as speed increased.

An increase in pulp cavity width had a decreased success rate in quality of fill with use of the Lentulo spiral filler. Depending on technique, operation of the Lentulo was either performed with a set 5 pumps throughout the pulp cavity or total 120 s at the apex. Due to the standardized operation, it is likely that teeth with larger pulp cavity widths would require longer action to distribute a greater volume of sealer cement. Clinically, in situations with large pulp cavities, a catheter is often used in place of a Lentulo to deliver sealer cement. Ideally, this study would have had narrower canals (<120 FAS) to better represent the clinical situation where a Lentulo spiral filler would be used.

In regard to time to fill the pulp cavity in the current study, while the association of time and tooth type was not statistically significant, longer time was noted for completion of pulp cavity fill at higher speeds, regardless of technique, and with the backfill technique, regardless of speed. Due to the finding that higher speeds have higher success in quality of fill, this confirms the possibility that longer filling time, especially in larger pulp cavity sizes, may have a correlation with quality of fill. However, further studies are needed to confirm this.

Limitations of the current study include small sample sizes for each group, relatively large pulp cavity sizes, and an *in vitro* study design. While a larger sample size may have strengthened results in the statistical analysis, it was important to have similar sized pulp cavities for comparing the different speeds and techniques of the Lentulo spiral filler, which limited the cadaveric specimens able to be utilized based on availability. Another limitation was the use of one method for evaluating voids throughout the canals, which was at risk of the effect of angulation change, introducing bias. CBCT was also performed post-oburation on these specimens, however severe artifacts were produced from the radiodense sealer cement, thus we were unable to use this methodology to effectively evaluate post-obturation void volume. Full review of the use of CBCT for evaluating endodontic therapy is out of the scope of this article. To the authors’ knowledge, there is no current standardization for measuring voids following obturation in veterinary or human literature. Future studies may benefit from utilizing other imaging modalities such as micro-CT.

Lastly, our study only evaluated the use of one sealer cement (an epoxy resin-based sealer), limiting conclusions on the Lentulo spiral filler technique with other sealer cements. Both epoxy resin-based and gutta percha-based sealers are commonly used in veterinary patients (12–14). This study cannot be directly applied to clinically guide Lentulo spiral filler use for gutta-percha-based sealers (GuttaFlow) which have different material properties. Further, the tested epoxy-resin based sealer has distinct limitations, primarily the inability to bind the dentin and truly “seal” the pulp chamber and cytotoxicity if extruded (15). Calcium silicate-based sealers are increasingly utilized in human dentistry due to the true sealing ability, bioactivity, and lower cytotoxicity (15, 38). These are likely the best sealer cement option clinically, and future cadaveric and clinical work should explore the best instrumentation techniques for this sealer class.

## Conclusion

Within the limitations of the current study, the results support the hypothesis that a Lentulo spiral filler operated at higher rotary speeds results in the higher quality of fill, with a speed of 2,000 RPM significantly associated with the highest success rate in quality of fill. Although not statistically significant, the pumping technique was associated with a higher probability of success following master GP cone placement as compared with the backfill technique. Homogeneity also increased with higher rotary speeds and fill was most successful in the apical section, which is most clinically impactful. While further studies are needed to support the current data, we recommend using the Lentulo spiral filler at speeds no lower than 2,000 RPM with the pumping technique to maximize clinical success when delivering epoxy resin-based sealer cement.

## Data availability statement

The original contributions presented in the study are included in the article/Supplementary material, further inquiries can be directed to the corresponding author.

## Ethics statement

Ethical approval was not required for the study involving animals in accordance with the local legislation and institutional requirements because teeth were used from canine cadavers that were euthanized for reasons unrelated to the study.

## Author contributions

HC: Writing – original draft, Writing – review & editing, Conceptualization, Investigation, Methodology, Project administration. JM: Writing – review & editing, Project administration. BL: Writing – review & editing, Project administration. AR: Writing – review & editing, Formal analysis. SG: Writing – review & editing, Conceptualization, Investigation, Methodology, Project administration.
