# Supplementary material for: Evaluation of the efficacy of the Lentulo spiral filler operated at four different speeds and with two different techniques in cadaveric canine teeth of dogs
Source: Front Vet Sci. 2023 Nov 2;10:1295306. doi: 10.3389/fvets.2023.1295306 (PMC10652754; doi:10.3389/fvets.2023.1295306)
Supplement: Supplementary file 1 [file Table_1.DOCX]

**Supplementary Material**

Supplementary Table 1: ANOVA models for each potential variable on quality of fill

| **Model Term** | **Degree of Freedom** | **Chisq** | **P-value** |
| --- | --- | --- | --- |
| Technique | 1 | 0.001 | 0.9773 |
| RPM | 3 | 24.867 | <0.0001 |
| Location | 2 | 33.832 | <0.0001 |
| Tooth Type | 1 | 3.741 | 0.0531 |
| Method | 1 | 10.874 | 0.0010 |
| Technique:Method | 1 | 4.49 | 0.0341 |
| RPM:Method | 3 | 15.747 | 0.0013 |
| Location:Method | 2 | 1.388 | 0.4997 |
| Tooth Type:Method | 1 | 0.684 | 0.4082 |
